# Risk of subsequently developing lower urinary tract symptoms in patients with plantar fasciitis: A nationwide, population-based study

**DOI:** 10.1097/MD.0000000000043349

**Published:** 2025-07-18

**Authors:** Yen-Lun Kung, Hsuan-Shu Shen, Li-Kung Wu, Wei-Chuan Chang, Hann-Chorng Kuo, Tsung-Jung Ho

**Affiliations:** a Department of Chinese Medicine, Hualien Tzu Chi General Hospital, Buddhist Tzu Chi Medical Foundation, Hualien, Taiwan; b Institute of Medical Sciences, Tzu Chi University, Hualien, Taiwan; c School of Post-Baccalaureate Chinese Medicine, Tzu Chi University, Hualien, Taiwan; d Epidemiology and Biostatistics Consulting Center, Department of Medical Research, Buddhist Tzu Chi General Hospital, Hualien, Taiwan; e Department of Urology, Hualien Tzu Chi Hospital, Buddhist Tzu Chi Medical Foundation, and Tzu Chi University, Hualien, Taiwan.

**Keywords:** cohort study, lower urinary tract symptom, meridian, plantar fasciitis, traditional Chinese medicine

## Abstract

Plantar fasciitis (PF) is rarely considered to be associated with lower urinary tract symptoms (LUTS). However, PF and LUTS are closely connected within the theory of traditional Chinese medicine and the myofascial meridian system. This study explored the association between PF and LUTS. We conducted a retrospective population-based cohort study using the Taiwan National Health Insurance Research Database. Patients with a diagnosis of PF <3 times within half a year, aged <18 years, or diagnosed with LUTS before the diagnosis of PF were excluded. After exclusion, we enrolled a non-PF group through propensity-matching (1:1) by age, gender, and prior lack of LUTS history. Multivariate Cox regression analysis revealed that patients with PF had an increased risk of LUTS (adjusted hazard ratio = 1.35; 95% confidence interval, 1.23–1.47) compared with non-PF patients after adjustment. Kaplan–Meier analysis indicated that the cumulative incidence of LUTS in the PF group was significantly higher than that in the non-PF group (*P* ≤ .001). Further analyses revealed that PF was an independent factor associated with an increased risk of developing LUTS in subgroups of both sexes, aged ≤50 years and >50 years, and with a follow-up period of ≤ 5 years and > 5 years. This is the first study demonstrating that patients diagnosed with PF have an increased risk of subsequently developing LUTS. Further research is needed to clarify the causal relationship between PF and LUTS.

## 1. Introduction

Plantar fasciitis (PF) is a common soft-tissue inflammatory disorder of the foot. The hallmark symptom is a sharp pain localized at the heel, especially in the first step in the morning or after a period of inactivity.^[1]^ It is estimated that up to 10% of active individuals experience PF over a lifetime.^[2]^ An increased risk of PF is highly associated with prolonged weight bearing, obesity, and advanced age, especially in the mid to late 40s.^[3]^ PF can become chronic if untreated or mismanaged, leading to further complications.^[4]^ A long-term study reported a recurrence rate of 55% in those receiving conservative treatments.^[5]^ Lower urinary tract symptoms (LUTS) describe a wide variety of storage, voiding, and post-micturition symptoms.^[6]^ The prevalence of LUTS ranged from 12.6 to 25.1%,^[7]^ increasing with age, both in men and women.^[8–10]^ Individuals aged over 60 years have an increased risk of suffering moderate to severe symptoms.^[11]^ Both PF and LUTS are known to negatively impact health-related quality of life and healthcare burden.^[6,12]^ In the past, no studies have linked PF and LUTS. However, these 2 seemingly unrelated diseases are closely connected within the framework of the traditional Chinese medicine (TCM) theory and the myofascial meridian system.

Several lines of circumstantial evidence suggest the relationship between PF and LUTS. Firstly, the distribution of the Kidney Meridian and the Bladder Meridian encompasses the pelvic cavity and the feet, particularly the muscles and fascia of the soles.^[13]^ According to the meridian theory in TCM, Qi and blood circulate through the meridians and nourish the tissues they pass by. If a meridian becomes stagnant, the related organs or tissues may become disordered and gradually develop a disease.^[14]^ Secondly, acupoints commonly used in TCM to treat PF and LUTS often overlap.^[15–18]^ In addition to targeting specific meridians, the selections of these acupoints are based on the syndrome differentiation theory in TCM, which identifies kidney deficiency syndrome as a common pathological mechanism underlying both diseases.^[19,20]^ Thirdly, the plantar fascia is supplied by the lateral and medial plantar nerves that arise from the posterior branch of the tibial nerve with root values of L4, L5, S1, S2, and S3. These fibers also supply the pelvic floor muscles and innervate the bladder and urinary sphincter. Particularly, percutaneous tibial nerve stimulation (PTNS) at approximately 3 to 4 cm above the medial malleolus has been widely used in managing LUTS, including overactive bladder (OAB) and voiding dysfunction. The location for the electrical stimulation coincides with the areas of pain and tightness associated with PF.^[21]^ Fourthly, it has been recently suggested that repetitive musculoskeletal pain can increase urinary afferent signaling above the normal threshold, resulting in urinary symptoms of urgency and frequency.^[22]^ These findings support the notion that central sensitization is a mechanism of LUTS under that setting.^[22]^

This study aimed to evaluate the impact of PF on the subsequent development of LUTS. To achieve this goal, we used a nationwide population-based dataset to conduct a propensity score-matched cohort study. Patients were classified as PF and non-PF for subsequent univariate and multivariate analysis.

## 2. Materials and methods

### 2.1. Data source

The National Health Insurance program, which was initiated in March 1995, covers 99% of the 23.7 million residents of Taiwan. The National Health Insurance Research Database was provided by the Data Science Center of the Ministry of Health and Welfare of Taiwan, and it contains comprehensive medical records including demographic characteristics, drug prescriptions, surgical procedures, and disease diagnosis codes from both outpatient and inpatient records.^[23]^ Their validity and accuracy have been demonstrated in previous studies.^[23]^ In this study, we used the Longitudinal Health Insurance Database 2000 (LHID 2000). LHID 2000 contains 2000,000 participants (approximately 10% of Taiwan’s population) randomly selected from National Health Insurance beneficiaries in Taiwan. The disease diagnostic codes were according to the International Classification of Diseases-Clinical Modification, 9th revision and 10th revision. Anatomical Therapeutic Chemical codes were used to recognize drug prescriptions. This study was approved by the Research Ethics Committee of Hualien Tzu Chi Hospital (approval number: IRB112-193-C).

### 2.2. Study cohort and design

**Figure 1.**
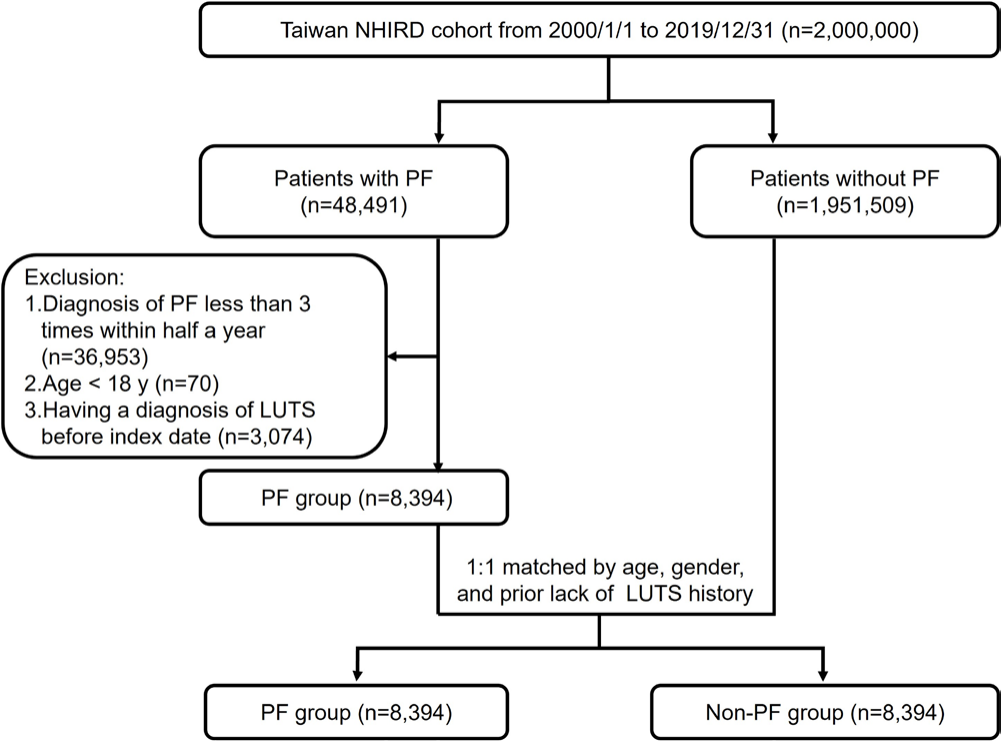
Study flowchart. NHIRD = National Health Insurance Research, PF = plantar fasciitis, LUTS = lower urinary tract syndrome.

We enrolled the patients diagnosed with PF by orthopedics or rehabilitation physicians from January 2000 to December 2019. In the PF group, the exclusion criteria were as follows: patients had a diagnosis of PF <3 times within half a year; patients aged <18 years at the time of PF diagnosis; and patients had a diagnosis of LUTS prior to the diagnosis of PF. After exclusion, the PF and non-PF groups were propensity-matched for age, sex, and prior lack of LUTS history to achieve a 1:1 study cohort (Fig. 1). We set the first date on which PF was diagnosed as the index date for the PF group, while the index date for the non-PF group was the date on which it was matched. The follow-up for patients began on the index date and lasted until withdrawal from the National Health Insurance program, until the diagnosis of LUTS, or on December 31, 2019, whichever occurred first.

### 2.3. Covariates and outcomes

We collected baseline demographic data, including age and sex. Comorbidities, including hypertension, diabetes mellitus, hyperlipidemia, depression, back pain, cerebrovascular diseases, urinary tract infection, and obesity, were identified through the ICD codes (Table S1, Supplemental Digital Content, https://links.lww.com/MD/P408). Baseline drug use, including diuretics, muscle relaxants and sedatives, antipsychotics, narcotics, antihistamines, and alpha-adrenergic antagonists, was identified by Anatomical Therapeutic Chemical codes (Table S2, Supplemental Digital Content, https://links.lww.com/MD/P408). The outcomes of this study were the events of LUTS, including difficult urination, urinary retention, urinary frequency, urgency, nocturia, and urinary incontinence, identified by the ICD codes (Table S1, Supplemental Digital Content, https://links.lww.com/MD/P408) occurring during the follow-up period.

### 2.4. Statistical analysis

The chi-square test and the independent *t* test were used to test the difference between the 2 study cohorts for categorical variables and continuous variables, respectively. The balance of the covariates was measured using standardized mean differences (SMDs); SMD ≤ 0.2 indicates a negligible difference between the 2 study cohorts. Univariate and multivariate Cox proportional hazard regression models were used to calculate crude hazard ratios (HRs) and adjusted HRs with 95% confidence intervals (CIs) after adjusting for potential confounders, including comorbidities and drug use. To estimate the cumulative risk of LUTS during the 20-year follow-up period, we employed the Kaplan–Meier method with significance based on the log-rank test. Further subgroup analyses were performed according to sex (male vs female), age (≤50 yr vs >50 yr), and follow-up period (≤5 yr vs >5 yr). All statistical analyses were performed with SAS software version 9.4 (SAS Institute, Cary) and Stata version 15 (StataCorp LLC, College Station). A 2-sided test with a *P* value < .05 was considered statistically significant.

## 3. Results

We identified 48,491 patients with a new diagnosis of PF during the study period. After exclusion, 8394 PF patients were identified. After propensity score matching, each study group (PF and non-PF) consisted of 8394 patients (Fig. 1). Table 1 shows demographic and baseline clinical characteristics of the 2 propensity-matched study cohorts.

**Table 1:** Demographic and baseline clinical characteristics of the 2 propensity-matched study cohorts (with and without plantar fasciitis).

	PF	Non-PF	SMD
	n = 8,394	n = 8,394	
Sex (%)			<0.001
Male	5380 (64.09%)	5380 (64.09%)	
Female	3014 (35.91%)	3014 (35.91%)	
Age, yr (%)			<0.001
Median (Q1, Q3)	52 (43, 60)	52 (43, 60)	
≤50	3795 (45.21%)	3795 (45.21%)	<0.001
>50	4599 (54.79%)	4599 (54.79%)	
Follow-up, yr			0.025
Median (Q1, Q3)	4.78 (2.23, 8.41)	4.83 (2.28, 8.57)	
≤5	4262 (50.77%)	4307 (51.31%)	0.011
>5	4132 (49.23%)	4087 (48.69%)	
Comorbidity (%)			
Hypertension	1853 (22.08%)	1536 (18.30%)	0.094
Diabetes mellitus	900 (10.72%)	822 (9.79%)	0.031
Hyperlipidemia	1049 (12.50%)	754 (8.98%)	0.114
Depression	303 (3.61%)	225 (2.68%)	0.053
Back pain	2020 (24.06%)	1125 (13.40%)	0.276
Cerebrovascular diseases	189 (2.25%)	226 (2.69%)	0.028
Urinary tract infection	584 (6.96%)	476 (5.67%)	0.053
Obesity	88 (1.05%)	50 (0.60%)	0.050
Drug (%)			
Diuretics	184 (2.19%)	199 (2.37%)	0.012
Muscle relaxants and sedatives	474 (5.65%)	423 (5.04%)	0.027
Antipsychotics	13 (0.15%)	32 (0.38%)	0.045
Narcotics	0 (0%)	3 (0.04%)	0.028
Antihistamines	126 (1.50%)	102 (1.22%)	0.024
Alpha-adrenergic antagonists	80 (0.95%)	69 (0.82%)	0.014

PF = plantar fasciitis, SMD = standardized mean difference.

An SMD < 0.2 indicates a negligible difference between the 2 study groups.

Except for the percentage of back pain, SMD in other covariates were <0.2, indicating a negligible difference between these 2 study cohorts (i.e., 2 well-balanced cohorts). The median ages for both the PF and non-PF groups were 52 years. The median follow-up times for the PF and non-PF groups were 4.78 and 4.83 years, respectively. Patients in the PF group had a significantly higher percentage of back pain at baseline (Table 1).

**Figure 2.**
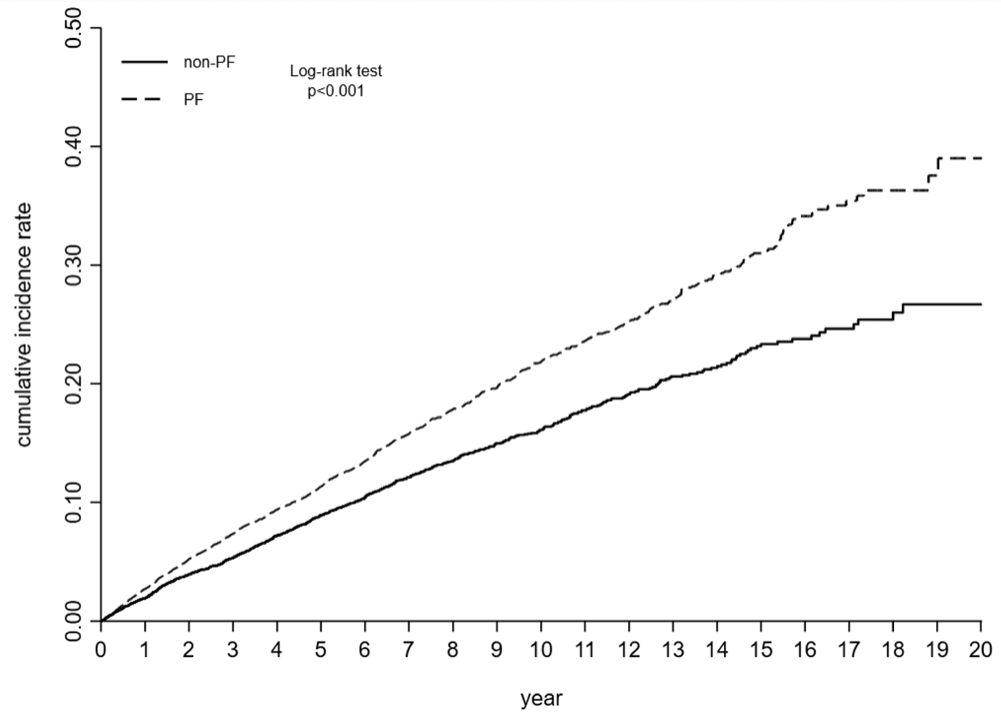
Kaplan–Meier curves showing the cumulative incidence rate of lower urinary tract symptoms in all study patients. PF = plantar fasciitis.

Table 2 shows the incidence rate and risk of developing LUTS in the whole group and subgroups stratified by sex, age, and follow-up period. As shown, the incidence rate of LUTS was 247.89 per 10,000 person-years in the PF group, and was 180.18 per 10,000 person-years in the non-PF group. The Cox proportional hazards model adjusted for characteristics in Table 1 revealed that patients with PF had a significantly higher risk of LUTS (HR = 1.35; 95% CI, 1.23–1.47) compared with patients without PF (Table 2). As shown in Figure 2, the cumulative incidence function demonstrated that the cumulative incidence of LUTS in the PF group was significantly higher than that in the non-PF group. Univariate Cox regression analysis revealed that PF patients had a significant increase in developing LUTS (crude HR [95% CI] = 1.38 [1.26–1.50]) compared with non-PF patients. Multivariate Cox regression analysis indicated that PF remained an independent factor associated with an increased risk of developing LUTS (adjusted hazard ratio [95% CI] = 1.35 [1.23–1.47]) after adjustment for confounding factors. The Kaplan–Meier curve analysis revealed that the cumulative incidence rate of LUTS in the PF group was significantly higher (Fig. 2, log-rank test, *P* < .001) than that in the non-PF group over the follow-up period.

**Table 2:** Incidence rate and risk of developing lower urinary tract symptoms in the whole group and subgroups stratified by sex, age, and follow-up period.

Characteristics	PF			Non-PF			PF vs non-PF HR (95% CI)	
	Event	PY	Rate	Event	PY	Rate	Crude	Adjusted
LUTS								
Whole group	1205	48610.67	247.89	892	49505.47	180.18	1.38 (1.26–1.50)	1.35 (1.23–1.47)
Sex								
Female	734	32483.27	225.96	568	32848.97	172.91	1.31 (1.17–1.46)	1.28 (1.14–1.43)
Male	471	16127.40	292.05	324	16656.50	194.52	1.50 (1.30–1.72)	1.45 (1.25–1.68)
Age								
≤50 yr	438	23487.56	186.48	303	24270.73	124.84	1.50 (1.29–1.73)	1.46 (1.25–1.69)
>50 yr	767	25123.11	305.30	589	25234.74	233.41	1.31 (1.17–1.46)	1.29 (1.15–1.43)
Follow-up								
≤5 yr	749	10129.54	739.42	578	10074.10	573.75	1.29 (1.15–1.43)	1.29 (1.15–1.44)
>5 yr	456	38481.13	118.50	314	39431.37	79.63	1.51 (1.31–1.74)	1.51 (1.31–1.75)

In the Cox proportional hazard model, adjusted HR was obtained after adjustment for comorbidity and drug use.

CI = confidence interval, HR = hazard ratio, LUTS = lower urinary tract symptoms, PF = plantar fasciitis, PY = person year, Rate = incidence rate, per 10,000 person-years.

**Figure 3.**
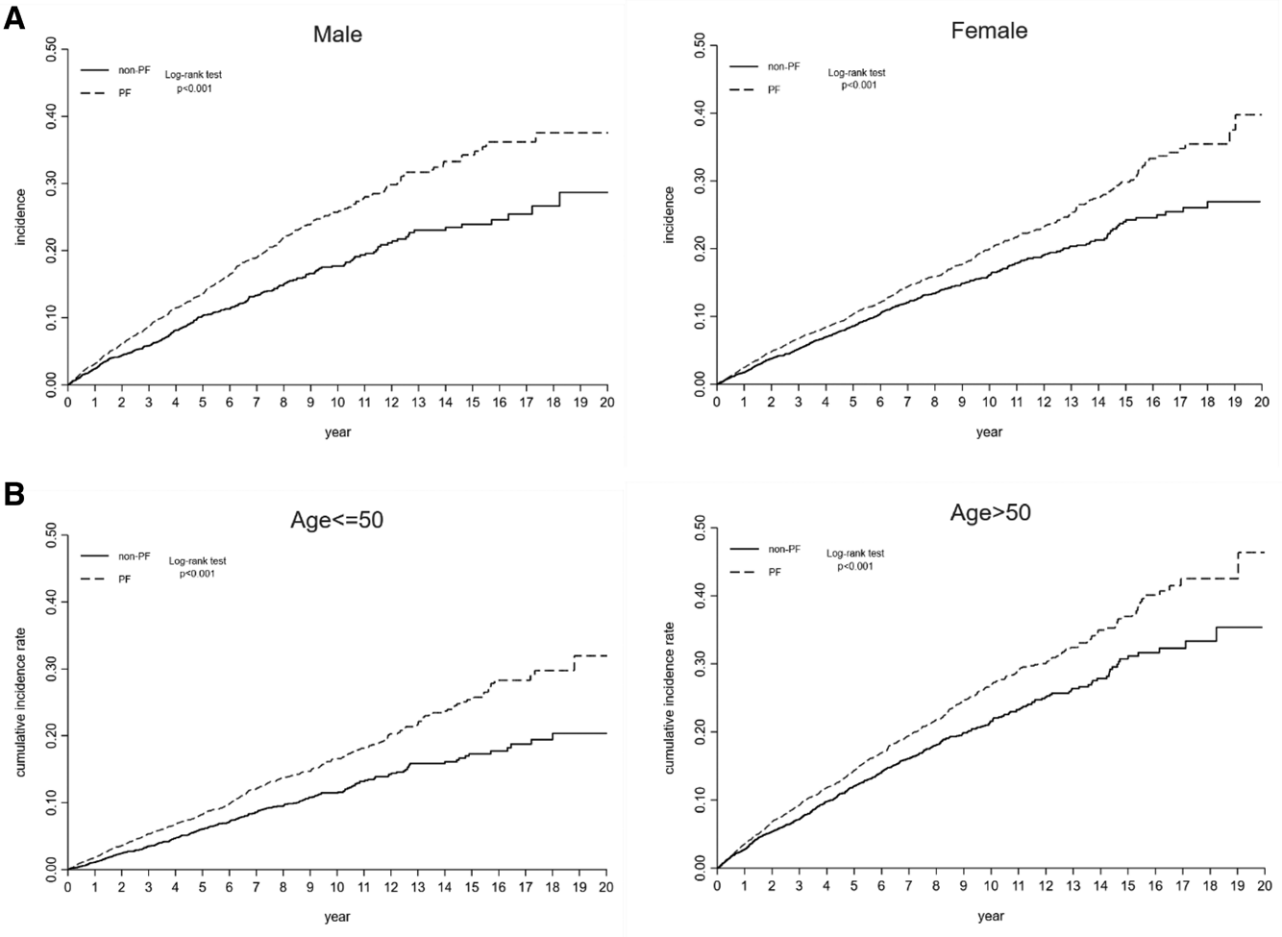
Kaplan–Meier curves showing the cumulative incidence rate of lower urinary tract symptoms in subgroups of patients. Subgroup analyses were stratified by (A) sex (male and female) and (B) age (≤50 yr and > 50 yr). PF = plantar fasciitis.

Subgroup analyses stratified by sex revealed that patients with PF had a significantly higher risk of LUTS in both females (HR = 1.28; 95% CI, 1.14–1.43) and males (HR = 1.45; 95% CI, 1.25–1.68). Subgroup analyses stratified by age revealed that patients with PF had a significantly higher risk of LUTS in both age groups of ≤ 50 years (HR = 1.46; 95% CI, 1.25–1.69) and of > 50 years (HR = 1.29; 95% CI, 1.15–1.43). The Kaplan–Meier curve analysis revealed that the cumulative incidence rate of LUTS in the PF group was significantly higher than that in the non-PF group over the follow-up period in these subgroups (Fig. 3; sex and age). Also, subgroup analyses stratified by the follow-up period revealed that patients with PF had a significantly higher risk of LUTS in both follow-up period ≤ 5 years (HR = 1.29; 95% CI, 1.15–1.44) and follow-up period > 5 years (HR = 1.51; 95% CI, 1.31–1.75).

## 4. Discussion

The major finding of this study is that patients with PF had an increased risk of subsequently developing LUTS based on a population-based propensity-matched cohort with 20 years of follow-up. Subgroup analysis suggested this association exists not only in males but also in females, suggesting no sex difference. PF had a higher prevalence in the late 40s,^[3]^ whereas individuals aged over 60 years may have an increased risk of moderate to severe LUTS.^[11]^ Therefore, we stratified the cohort at 50 years of age and observed that both subgroups (>50 yr and ≤50 yr) with a history of PF had an increased risk of subsequently developing LUTS. Additional subgroup analyses stratified by the follow-up period of 5 years (≤5 yr and >5 yr) revealed that this association remained present in both subgroups. PF tends to develop earlier than LUTS.^[3,11]^ Thus, the subsequent occurrence of LUTS following PF has important clinical implications, as it may guide preventive strategies and early interventions for patients.

The exact link between PF and the development of LUTS remains largely unclear. However, several lines of circumstantial evidence support the notion that PF is associated with LUTS. In TCM theory, meridians are channels through which Qi and blood circulate, nourishing the tissues they pass by. The kidney-bladder distinct meridian is traditionally recognized as the acupuncture channel most effective in treating genitourinary disorders. The distribution of the kidney meridian and the bladder meridian encompasses the pelvic cavity and the feet, particularly the muscles and fascia of the soles.^[13]^ If a meridian becomes stagnant, the related organs or tissues may become dysfunctional and gradually develop a disease.^[14]^ Syndrome differentiation is another theory used in TCM clinical practice. Kidney deficiency is considered a core pathological basis shared by both heel pain and LUTS.^[19,20]^ Therefore, in the treatment of PF, acupoints such as Chengshan (BL 57), Kunlun (BL 60), Taixi (KI 3), and Sanyinjiao (SP 6) are commonly selected for treating renal and urinary tract diseases, including LUTS.^[15–18]^

According to myofascial meridian theory, there are also certain connections between the foot and pelvic structure, especially the superficial back line and the deep front line.^[24]^ The superficial back line connects the plantar fascia, long plantar ligament, sacrum, and sacrotuberous ligament. The deep front line forms the core of the body’s myofascial system, linking the flexor hallucis longus, tibialis posterior, sacrum, pelvic floor muscles, puborectalis muscle, and levator ani muscle.^[24]^ Pelvic floor muscles are innervated by both visceral and somatic nerves, including afferent and efferent nerves, that also supply the bladder.^[25]^ Disturbed proprioceptive signals of pelvic floor muscles due to increased muscle tension may lead to LUTS caused by uncontrolled sensory signals originating from the stretch receptors continuously provoke premature stimulation of the micturition reflex.^[26]^

In clinical practice, it has been observed that LUTS might have an association with tension in the lower extremity and pelvic floor muscles. Peters et al reported that, in patients with interstitial cystitis, 87% had levator pain with concomitant pelvic floor dysfunction.^[27]^ Hyperactive pelvic floor muscles cause dysfunctional voiding and functional bladder outlet obstruction, leading to the development of LUTS.^[28]^ On the other hand, pelvic floor muscle training has been widely used in the treatment of OAB and urinary incontinence.^[29]^ Moreover, the tension of the leg may influence the normal function of pelvic floor muscles. Pires et al reported that the maximum voluntary contraction values of pelvic floor muscles have a negative correlation with urinary symptoms, and these values decrease with increasing leg stiffness;^[30,31]^ this observation adds evidence to support the functional connection between the lower extremity and pelvic floor fascia.

It has been reported that repetitive musculoskeletal pain was associated with urinary symptoms of urgency and frequency, and central sensitization was proposed to be the underlying mechanism.^[22]^ Persistent peripheral nociceptive input can lead to increased excitability of neurons within the dorsal horn of the spinal cord and higher centers of the central nervous system.^[32]^ This neuroplastic change may result in nonspecific pain amplification and cross-organ sensitization.^[32]^ In our study, PF, as a form of chronic musculoskeletal pain, may induce such central sensitization through sustained afferent stimulation originating from the foot. Given that the sensory innervation of the foot and the lower urinary tract partially overlaps at the spinal segmental level (e.g., L5–S2), prolonged nociceptive signaling from PF may dysregulate sensory processing in neighboring pelvic organs. This aberrant sensory processing may lead to enhanced perception of normal bladder distension as noxious, thereby contributing to the development of LUTS such as urgency, frequency, and nocturia.^[33]^ Collectively, central sensitization may be a potential mechanism linking PF and LUTS.

In clinical practice, there is substantial overlap in the treatment strategies for PF and LUTS. Electrical stimulation has been developed as the second- or third-line option in treating symptoms of LUTS, and is well validated nowadays.^[34]^ Currently, PTNS is the least invasive form of neuromodulation approved by the Food and Drug Administration for treating OAB, urge incontinence, and urinary frequency incontinence.^[35]^ The mechanism of PTNS is still unclear. Some studies suggest a supraspinal effect or retrograde stimulation of the sacral nerve plexus since the posterior tibial nerve is derived from the L4-S3 nerve roots and therefore shares common roots with those having bladder functions.^[36]^ Technically, electrodes were placed 5 cm above the medial malleolus, applied to the sole of the foot.^[37]^ These locations overlap the sites of pain and tightness in patients with PF, and are commonly used points to alleviate the pain associated with PF in clinical practice. Recently, ultrasound-guided pulsed radiofrequency stimulation of the posterior tibial nerve provides a potential novel intervention for recalcitrant or intolerable PF.^[38]^

This study has some limitations. First, our retrospective study may have several biases, including data collection and the differences between the 2 study cohorts. We believed that these biases could be minimized by the study design using propensity-matched analyses. However, covariates such as disease severity, smoking, lifestyle, and education may be related to the development of LUTS, but are not registered in the health insurance database. Thus, unmeasured differences between these 2 cohorts may still account for our observed results. Second, LUTS refers to a group of symptoms that can occur in various diseases with different pathophysiology, such as benign prostatic hyperplasia, neurogenic lower urinary tract dysfunction, detrusor underactivity, OAB, and stress urinary incontinence. Thus, our finding regarding the relationship between PF and LUTS cannot be attributed to any specific disease. Third, all patients were Taiwanese individuals, and the generalizability of the findings to other races or patients is limited. Fourth, this study involved cohort-matching processes that inevitably excluded participants whose matching covariates did not qualify to be included in the analyzed cohort. Our findings should not be generalized to all patients.

## 5. Conclusion

In conclusion, this is the first study demonstrating the relationship between PF and LUTS. Compared to subjects without PF, patients with a previous diagnosis of PF were more likely to develop LUTS. Further research is needed to clarify the causality between PF and LUTS.

## Acknowledgments

We thank Prof Yu-Ru Kou, Dr Yi-Chen Chen, Dr Jia-Fong Jhang, and Dr Huei-Kai Huang at Hualien Tzu Chi General Hospital for their advice during the research.

## Author contributions

**Conceptualization:** Yen-Lun Kung, Hsuan-Shu Shen, Li-Kung Wu, Hann-Chorng Kuo, Tsung-Jung Ho.

**Data curation:** Yen-Lun Kung, Hsuan-Shu Shen, Li-Kung Wu, Wei-Chuan Chang.

**Formal analysis:** Yen-Lun Kung, Li-Kung Wu, Wei-Chuan Chang.

**Investigation:** Yen-Lun Kung, Li-Kung Wu, Wei-Chuan Chang.

**Methodology:** Yen-Lun Kung, Hsuan-Shu Shen.

**Writing – original draft:** Yen-Lun Kung, Hsuan-Shu Shen, Wei-Chuan Chang.

**Writing – review & editing:** Hann-Chorng Kuo, Tsung-Jung Ho.

## Supplementary Material


