# Supplementary material for: Risk of subsequently developing lower urinary tract symptoms in patients with plantar fasciitis: A nationwide, population-based study
Source: Medicine (Baltimore). 2025 Jul 18;104(29):e43349. doi: 10.1097/MD.0000000000043349 (PMC12282792; doi:10.1097/MD.0000000000043349)
Supplement: Supplementary file 1 [file medi-104-e43349-s001.docx]

**Supplemental Content**

**Risk of** **subsequently developing lower urinary tract symptoms in patients with plantar fasciitis: a nationwide, population-based study**

First author: Yen-Lun Kung

Table 1 Diagnostic for covariates

|  | **ICD-9-CM codes** | **ICD-10-CM codes** |
| --- | --- | --- |
| Plantar fasciitis | 728.71 | M72.2 |
| Hypertension | 401-405 | I10-I16 |
| Diabetes mellitus | 250 | E08-E13 |
| Hyperlipidemia | 272.4 | E78.4, E78.5 |
| Depression | 296.2, 296.3, 300.4, 309.0, 309.1, 311 | F32, F33, F34.1, F43.21 |
| Back pain | 724 | M43.2, M48.00, M48.04-M48.08, M51.1, M53.3, M53.80, M53.84-M53.88, M53.9, M54.03-M54.09, M54.14-M54.17, M54.3-M54.6, M54.9, M99.22-M99.29, M99.32-M99.39, M99.42-M99.49, M99.52-M99.59, M99.62-M99.69, M99.72-M99.79 |
| Cerebrovascular diseases | 430-438 | I60-I69 |
| Urinary tract infection | 599.0 | N39.0 |
| Overactive bladder | 596.51 | N32.81 |
| Lower urinary tract symptoms | 596.0, 600, 625.6, 788.1-788.4, 788.6 | R30, R32, R33, R35, R39 |
| Obesity | 278, 649.2, 783.1, V45.86, V77.8, V85.2-V85.4 | E65-E68, R63.5, Z13.89 |

ICD-9-CM = International Classification of Disease-Clinical Modification, 9th revision, ICD-10-CM = International Classification of Disease-Clinical Modification, 10th revision.

Table 2 Anatomical Therapeutic Chemical codes for baseline drug prescriptions

|  | **ATC codes** |
| --- | --- |
| Diuretics | C03AA03, C03EA01, C03CA01, C03CA02 |
| Muscle relaxants and sedatives | N05BA01, N05BA02, N05BA06 |
| Antipsychotics | N05AA01, N05AC02, N05AF03, N05AB06, N05AB02, N05AD01, N05AG02 |
| Narcotics | N02AA05, N02AA01 |
| Antihistamines | R06AA02, R06AB04 |
| Alpha-adrenergic antagonists | G04CA03, C02CA04 |

ATC = Anatomical Therapeutic Chemical
